# Mitral Transcatheter Edge-to-Edge Repair in Non-Surgical Candidates with Hypertrophic Obstructive Cardiomyopathy: Clip It, or Ablate It?

**DOI:** 10.3390/jcdd13060255

**Published:** 2026-06-08

**Authors:** Emmanouil Chourdakis, Kambis Mashayekhi, Ulrich Schäfer, Christos Katsouras

**Affiliations:** 1 Internal Medicine and Cardiology, Heart Center Lahr, 77933 Lahr, Germany; kambis.mashayekhi@mediclin.de; 2 First Department of Cardiology, Faculty of Medicine, School of Health Sciences, University of Ioannina, 45110 Ioannina, Greece; cskats@yahoo.com; 3 First Department of Cardiology, Faculty Heart and Vascular Centre, 29549 Bad Bevensen, Germany; u.schaefer@hgz-bb.de

**Keywords:** hypertrophic obstructive cardiomyopathy, mitral regurgitation, TASH, M-TEER

## Abstract

Hypertrophic cardiomyopathy (HCM), with or without obstructive phenomena, remains underdiagnosed and undertreated. This condition often involves pathological changes in the mitral valve leaflets and apparatus, which can lead to relevant mitral regurgitation (MR). The mechanism of MR is mostly related to the systolic anterior motion (SAM) of the anterior mitral leaflet. The treatment of patients with hypertrophic obstructive cardiomyopathy (HOCM) with persistent symptoms despite optimal pharmacological therapy includes septal myectomy or transcoronary ablation of septal hypertrophy (TASH). Percutaneous edge-to-edge repair of the mitral valve represents an innovative alternative therapy with promising results regarding clinical symptoms and echocardiographic findings. In this article, we provide a concise, critical overview of the current evidence on this technique in HOCM and delineate future perspectives and unresolved issues.

## 1. Introduction

Hypertrophic cardiomyopathy (HCM) is an inherited cardiomyopathy with a prevalence of approximately 1:500 and is characterized by left ventricular hypertrophy, typically diagnosed using two-dimensional echocardiography or cardiac magnetic resonance imaging [1]. A subgroup of HCM patients presented with a dynamic left ventricular outflow tract (LVOT) obstruction, at rest in 30% and after provocation in an additional 30% [2,3]. The most common symptoms of HCM patients are dyspnea, syncope, and palpitations, although malignant arrhythmia leading to sudden cardiac death can be the first manifestation of the disease [4]. The subtype of HCM with obstructive phenomena has a higher rate of heart failure progression compared with the non-obstructive subtype [4]. 

In symptomatic patients with obstructive HCM, the European Society of Cardiology guidelines recommend, as first-line treatment (I Class, B Level), a non-vasodilating beta-blocker or verapamil (in patients who are intolerant to or have contraindications to beta-blockers), or, in addition, disopyramide [5]. In patients with persistent symptoms despite the maximum tolerated doses, the addition of mavacamten (a cardiac myosin ATPase inhibitor) is recommended (IIa Class, A Level) [5]. Based on current guidelines, surgical myectomy or transcoronary ablation for septal hypertrophy (TASH) (I Class, B Level) is considered the preferred invasive septal reduction therapy for patients with hypertrophic obstructive cardiomyopathy (HOCM) who remain symptomatic [5,6].

**Figure 1:**
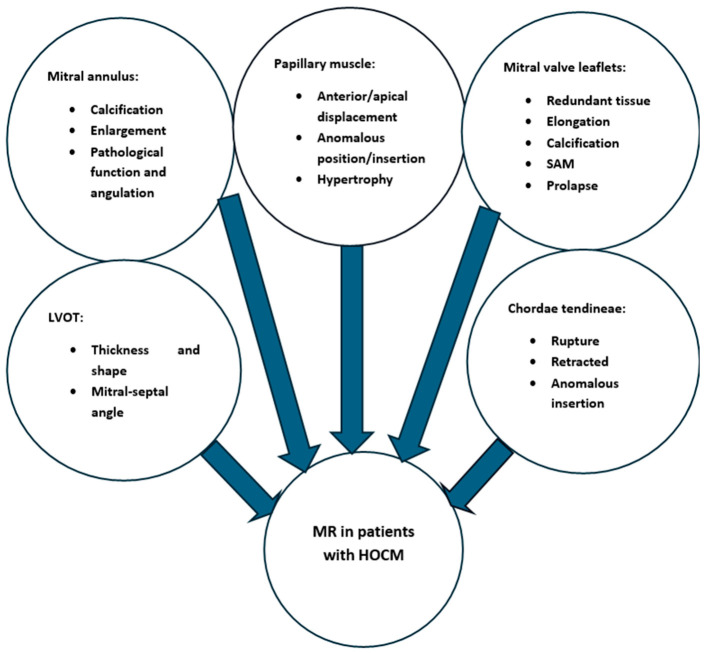
Different pathomechanisms of MR in patients with HOCM. The mitral valve, including the mitral annulus, chordae tendineae, papillary muscles, and leaflets, shows changes in patients with HCM compared with the normal population. In particular, leaflet redundancy arises from excess tissue and leaflet elongation. Degeneration of the leaflets with signs of calcification and prolapse can also be documented. Another interesting finding in HCM patients is elongation, hypertrophy, and apical displacement of the papillary muscles. Mitral annular dilatation, calcification, or pathological angulation are also noted. These alterations of the mitral valve apparatus, in combination with LVOT hypertrophy and the overall shape, as well as the mitral–septal angle, are important factors related to the exacerbation of SAM and MR severity. HOCM: hypertrophic obstructive cardiomyopathy, LVOT: left ventricular outflow tract, MR: mitral regurgitation, SAM: systolic anterior motion.

Mitral regurgitation (MR) is commonly observed in patients with HOCM (Figure 1). In most cases, MR results from the secondary to systolic anterior motion (SAM) of the anterior mitral leaflet, which is associated with dynamic outflow tract obstruction and is typically characterized by a posteriorly directed jet [7]. However, MR in HOCM is not solely associated with SAM, and other mechanisms may also be involved. The presence of more centrally or anteriorly directed regurgitation jets should be a red flag, indicating additional mitral valve pathology beyond SAM [7]. The mitral valve apparatus and its substructures, such as anterior displacement of the papillary muscles, abnormal secondary chordae attachment to the anterior mitral leaflet, and elongated redundant leaflets, can contribute to failed TASH and promote further LVOT obstruction [8,9]. Moreover, significant primary MR caused by mitral valve chordal rupture or prolapse, as a concurrent mitral valve disease in HOCM patients, is a rare finding, occurring in 1% of the HCM population. This should not be overlooked and may require a completely different interventional approach [10]. Mitral annular shape, size, angulation, and function can also be assessed by 3D echocardiography and cardiac magnetic resonance imaging in patients with HOCM [11,12,13]. These patients exhibit mitral annular dilatation and reduced fractional shortening compared to control subjects. Moreover, posterior mitral annulus or leaflet calcification is a common finding in patients with HOCM due to mechanical stress on the mitral annulus [11,12,13].

The mitral transcatheter edge-to-edge repair (M-TEER) in HOCM patients represents an area of growing interest, particularly for patients who are not candidates for established septal reduction therapies and who have failed TASH or surgical myectomy. Our manuscript is descriptive rather than analytical. It reflects the limited number of randomized studies and aims to evaluate the emerging role of this treatment while identifying gaps in the current evidence, especially in comparison with established septal reduction therapies. Nevertheless, we have detailed this topic and proposed an algorithm that we believe will be valuable for cardiologists and interventionalists who are exploring this approach.

## 2. Mitral Transcatheter Edge-to-Edge Repair in HOCM Patients

M-TEER is a well-established treatment for mitral valve disease [14]. According to guidelines from the European Society of Cardiology, M-TEER should be considered in selected symptomatic patients with chronic severe atrial secondary MR who are not eligible for surgery, despite optimal medical treatment and meeting the echocardiographic eligibility criteria, with II B level, class B [15].

A new off-label application of M-TEER may be for patients with HOCM. In 2014 Schäfer and colleges described for the first time a meaningful reduction of LVOTO related to SAM after M-TEER [15,16]. Unfortunately, the number of cases and long-term outcome data on the use of M-TEER as an interventional approach in HOCM are limited. The narrowing of the LVOT mainly depends on the degree of septal hypertrophy, but it is also influenced by the mitral valve apparatus, particularly the redundant anterior mitral leaflet and its abnormal movement. In HOCM, M-TEER may functionally mimic surgical anterior leaflet plication by restricting leaflet mobility and shifting the coaptation zone posteriorly, thereby reducing SAM and LVOT obstruction. In our view, M-TEER might be a safer or more effective option for patients who are poor surgical candidates, either as a stand-alone alternative or as an adjunctive therapy to TASH. The patient’s anatomical suitability for percutaneous edge-to-edge repair is a key factor when considering M-TEER as an alternative treatment option in patients with HOCM.

The disadvantages of TASH, compared to M-TEER, include the risk of cardiac conduction abnormalities that may require a pacemaker or lead to life- threatening arrhythmias or even sudden cardiac death, especially on the substrate of infarcted myocardium [17,18]. The risk of complete atrioventricular block (AV block) after TASH is 10–20%, and one in three patients will develop right bundle branch block (RBBB) [19,20]. A newly detected RBBB in a patient with pre-existing left bundle branch block (LBBB) can significantly increase the risk of complete cardiac block. Clip-associated conduction abnormalities are rare in non-HCM patients with severe MR. Eggebrecht et al. reported a 0.2% incidence of permanent pacemaker implantation following MitraClip, without detailed information on the underlying indications [21]. In a meta-analysis, Wang et al. found no significant need for pacemaker implantation, whereas Schnitzler et al. did not consider it a noteworthy complication [22,23]. Obviously, there are no relevant details in HOCM patients after M-TEER procedures. If data support that M-TEER can be performed in these patients without the need for a permanent pacemaker, it would be reasonable to consider the method as a first-line approach instead of TASH for patients at risk of pacemaker implantation. Additionally, in non-HCM patients, sudden cardiac death or life-threatening ventricular arrhythmias appear to be uncommon after MitraClip implantation, based on a systematic review by Cameron et al. published in Heart, Lung and Circulation [24]. A meta-analysis of five studies involving patients undergoing MitraClip who reported ventricular arrhythmia rates before and after the procedure showed a significant reduction in ventricular arrhythmias, non-sustained VT, and VT/VF [24]. These findings support the hypothesis that correcting MR, which reduces mechanical cardiac stress, may help lower the risk of sudden cardiac death [24]. Nevertheless, there remains a residual risk of life- threatening arrhythmias, largely influenced by patient-specific factors such as advanced age, reduced ejection fraction, elevated EuroScore, end-stage renal disease, pulmonary hypertension, and atrial fibrillation [25]. Importantly, current evidence on ventricular arrhythmias following MitraClip is limited, underscoring the need for larger, well-designed studies. Clearly, there is no available data on the risk of SCD in HOCM patients after MR repair with M-TEER. LVOT thickness for TASH is generally considered to be greater than 17 mm to ensure a safe procedure and minimize the risk of iatrogenic ventricular septal rupture. In contrast, basal septal thickness greater than 25 mm is a predisposing factor for TASH failure [26,27]. Septum hypertrophy measuring about 15–17 mm is a more borderline anatomy, indicating the need for alternative septal reduction therapy [26,27]. The inability to identify a target septal branch occurs in up to 10% of TASH patients and should also be considered an important factor in procedural planning [28]. From our perspective, unsuitable LVOT geometry, coronary septal branch anatomy, or pre-existing bundle branch block make M-TEER a more favourable therapeutic strategy, especially in patients with SAM. It should be highlighted that M-TEER is more appropriate for patients with significant MR who present with a more dynamic LVOTO, than for patients with LVOT narrowing due to significant left ventricular hypertrophy.

Another important aspect is the management of HOCM patients after failed TASH. Redo TASH is required in approximately 6.6% of patients, and surgical myectomy is performed in 1.9% to address persistent symptoms [29,30]. Importantly, perioperative mortality of surgical myectomy after failed TASH is substantially higher (6%) than in matched patients undergoing septal myectomy as the first and only treatment [31]. In this challenging clinical setting, M-TEER applied within the SESAME (septal reduction by edge-to-edge repair and systolic anterior motion elimination) concept may represent a potential alternative for carefully selected patients who are not candidates for redo TASH, while avoiding the need for high-risk surgical myectomy [32,33]. However, SESAME is a novel, technically demanding approach, currently supported only by limited case-based experience, and should therefore be restricted to rare, highly selected HOCM patients treated at specialized expert centers. In addition, in the presence of concomitant primary mitral valve disease, an M-TEER-based strategy may allow simultaneous treatment of both pathomechanisms, namely SAM-related LVOT obstruction and primary MR, within a single procedure. Therefore, M-TEER may be a game changer for patients with failed TASH who are ineligible for redo TASH, while also avoiding the need for high-risk procedures like surgical myectomy or SESAME after failed TASH. Furthermore, the presence of relevant concomitant primary mitral valve disease can be considered when choosing M-TEER over TASH as a first-line treatment, thereby targeting both pathomechanisms in a single procedure: first, SAM with LVOT obstruction; and second, the component of primary MR.

**Figure 2:**
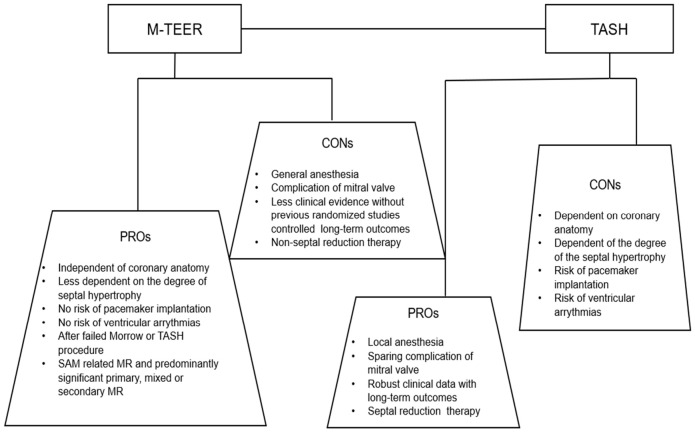
M-TEER versus TASH in poor surgical HOCM candidates despite optimal medical treatment. M-TEER: mitral transcatheter edge to edge repair, MR: mitral regurgitation, SAM: systolic anterior motion, TASH: transcoronary ablation of septal hypertrophy.

In conclusion, the following clinical factors may even support considering M-TEER instead of TASH as a primary therapeutic option (Figure 2): 1. lower risk of conduction abnormalities or pacemaker implantation, especially in high-risk patients with previous LBBB; 2. suitability for patients with septal hypertrophy of less than 15–17 mm; 3. patients who lack a suitable septal branch or have unfavorable LVOT geometry for TASH; 4. presence of relevant concomitant primary mitral valve disease; or 5. previous unsuccessful TASH.

## 3. Clinical Evidence for M-TEER in HOCM

Schäfer and colleagues reported, for the first time, the successful use of M-TEER in HOCM patients in 2014 and 2015. They treated three HOCM patients with MitraClip, resulting in improvements in rest peak gradients (before M-TEER: 65 ± 25.5 mmHg; after M-TEER: 7.7 ± 5.0 mmHg) and provoked pressure gradients (before M-TEER: 145.3 ± 8.1 mmHg; after M-TEER: 23.2 ± 7.6 mmHg) [16]. One of these patients had previously undergone a Morrow operation and mitral valve reconstruction, and another had previous TASH. The clinical and hemodynamic improvements were observed at a six-week follow-up [16]. These initial findings supported M-TEER as a viable option after failure of standard septal reduction therapies. Additionally, Kimmelstiel et al. observed a reduction in cardiac workload, assessed by pressure–volume loops, after successful M-TEER intervention in two patients with symptomatic HOCM who were not candidates for standard septal reduction therapies [34].

Sorajja et al. reported early success in HOCM patients who underwent MitraClip for symptom relief, MR, and LVOT reduction, with consistent results observed during the follow-up period (10–19 months) [35]. Eight years ago, Thomas et al. analyzed the beneficial role of M-TEER improving symptoms and hemodynamics in 15 HOCM patients with SAM across four studies [36]. Generally, M-TEER was adopted as the first-line strategy, with only one patient having failed a previous surgical myectomy and another failing an ASA attempt [36]. He also demonstrated elimination of SAM, a reduction in MR grade, and a significant decrease in the LVOT gradient from a mean of 75.8 ± 39.7 to 11.0 ± 5.6 mm Hg. After reviewing these two publications, M-TEER represents a valuable ‘arrow’ in our ‘quiver’ as a first-line approach [36]. 

Recently, Mascarenhas et al. published a patient-level meta-analysis after reviewing nineteen publications involving 37 HCM patients who underwent edge-to-edge repair of the mitral valve [37]. M-TEER not only reduced LVOT obstruction phenomena (the mean peak resting and provoked LVOT gradient: 69.2 ± 40.3 mmHg vs. 11.7 ± 8.6 mmHg and 98.2 ± 53.4 mmHg vs. 14.1 ± 13.9 mmHg, respectively) but also decreased the median MR grade (4.0 [3.0–4.0] vs. 1.0 [1.0–1.0]) [37]. The echocardiographic improvements were accompanied by a clinical improvement in NYHA functional class, from 3 to 1 (post-TEER: 100% vs. 7%; all *p* < 0.001) [37].

The decision to perform M-TEER versus TASH or myectomy, based on the evidence of isolated cases in Table 1, was supported by the clinical conditions of the patients (such as previous aortocoronary bypass surgery and stage IV cirrhosis), anatomical features (including prior TASH, Morrow operation, or jailed septal branch with stent), and the decision of the heart team, considering high perioperative risk, comorbidities, increased age, and frailty. After analysing the baseline characteristics (Table 1), most patients had severe symptomatic MR (NYHA III) with concurrent SAM, despite being on negative inotropic pharmacologic treatment. The median age was 71 years. Six of the 29 patients (20%) had previously undergone a Morrow or TASH attempt. A subgroup analysis comparing outcomes, pooled insights, and complications is unattainable due to the small number of cases and varying baseline characteristics, as the data are predominantly from case reports. In our view, the manuscript highlights that M-TEER was safe, feasible, and effective without major complications. A deeper subgroup analysis would still not provide properly powered data or reliable results. According to published data, most patients with HOCM underwent MitraClip implantation rather than the PASCAL device. Possible reasons for preferring the MitraClip system include its broader worldwide availability and longer data history, as well as more extensive anterior-to-posterior mitral leaflet plication, causing more leaflet tension. Most patients treated with MitraClip showed clinical improvement, along with echocardiographic reduction in MR and a decrease in the resting LVOT gradient during follow-up (Table 2). Extensively, the MR was quantified as trace, mild, or moderate in 28 of 29 patients, with only one patient having moderate to severe MR postprocedurally. Recurrence of MR occurred in only two cases. Regarding high-grade AV block or life-threatening arrhythmias, the rate was zero. No severe procedure-related complications were observed. LVOT gradients after the procedure were significantly reduced and remained consistently low on follow-up. Particularly, three cases had gradients above 20 mmHg and below 50 mmHg, and only two patients experienced extreme high gradients. In seven cases, no data were stated. The remaining patients, about 17, showed very low LVOT gradients.

The manuscript summarizes isolated cases or small case series in our Table 1 and Table 2. It is very challenging to compare individual cases and discuss biases due to the absence of randomized studies. Clearly, the current critical appraisal is difficult and limited to isolated cases. Designing randomized studies that compare the two strategies—M-TEER versus TASH—in HOCM patients who are not surgical candidates would be helpful. Such studies would raise the level of evidence and clarify the roles and scopes of each approach.

## 4. Selection of Percutaneous Strategies for HOCM Patients with MR

The percutaneous edge-to-edge repair of the mitral valve appears to be an alternative treatment option for patients who are not candidates for surgery or belong to a high surgical-risk group, especially those with a significantly increased LVOT gradient and SAM, and favorable anatomy for classical TASH treatment. 

The hemodynamic success rate after TASH is reported to be 70%, with 20% of those patients experiencing severe symptoms due to residual obstruction, which may require another septal reduction therapy in the range of 15–18% [32,50]. The re-operation rate following septal myectomy, as documented below, is under 2% [50]. Valeti et al. also showed that up to 25% of patients undergoing TASH had a residual LVOT gradient on postprocedural cardiac magnetic resonance imaging, attributable to sparing of the basal septum [51]. At this point, the possibility of redoing the TASH or SESAME based on echocardiographic and anatomical criteria should be considered, as should the determination of which component—SAM-associated MR or the LVOT gradient—is primarily responsible for the patient’s symptoms.

HOCM patients can be classified by the severity of obstruction at rest or after provocation, with this classification correlating with long-term cardiovascular events [52]. Patients with resting or provoked LVOT gradients above 30 mmHG, or resting LVOT gradients below 30 mmHg but a gradient of ≥90 mmHG after provocation, are considered a high-risk cohort with clear clinical therapeutic implications [53]. Furthermore, MR severity in HOCM patients should be thoroughly quantified based on echocardiographic criteria before decision-making, with initial treatment of MR using M-TEER against TASH [52,53]. It is questionable whether the standard criteria [53], with an effective regurgitant orifice area ≥0.4 cm^2^, an MR volume of 60 mL, and a regurgitant fraction above 50% define severe MR, as opposed to moderate MR, which is characterized by an effective regurgitant orifice between 0.2–0.39 cm^2^, MR volume of 30–59 mL, and a regurgitant fraction between 30–49%, should be applied for the utilization of M-TEER against TASH [53]. In cases with a proportionate MR (moderate to severe) relative to the degree of LVOT gradient (at least 30 mmHG at resting or more than 90 mmHg after provocation) and the presence of a septal branch as an additional target, current literature would support a redo TASH over M-TEER. Conversely, a disproportional relationship between significant MR (severe) and the LVOT gradient (maximal 30 mmHg or provoked gradient <90 mmHg) favours adopting M-TEER rather than redoing TASH. Diagnostic assessment can be quite complex in most patients because LVOT obstruction is dynamic (often related to SAM). The cornerstone is to detect signs of dynamic LVOT obstruction, especially using high positive Brockenbrough–Braunwald–Morro signs.

**Figure 3:**
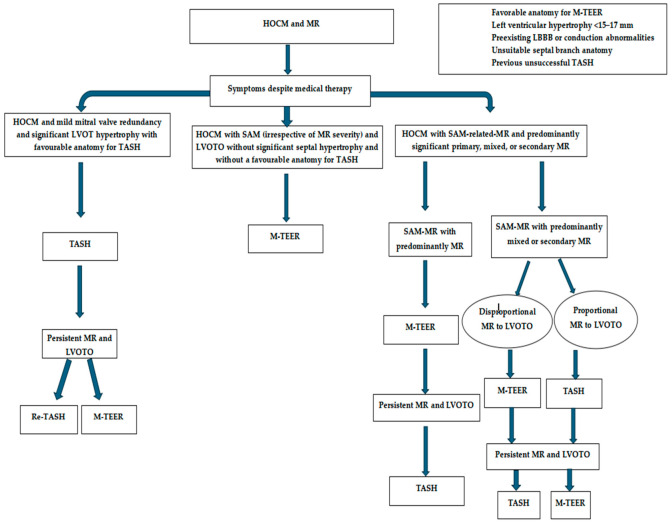
Percutaneous approach of non-surgical candidates with obstructive hypertrophic cardiomyopathy and concomitant mitral regurgitation.

We propose dividing HOCM patients into three most commonly documented scenarios (Figure 3), which are as follows:

A. HOCM and mild mitral valve redundancy and significant LVOT hypertrophy with favourable anatomy for TASH

This type of obstruction almost always occurs with midcavity obstruction (also called midventricular obstruction) and is reported in 10–15% of patients with HCM. TASH remains the preferred approach in symptomatic patients despite optimal medical therapy, when coronary and septal anatomy are favourable, as recommended by guidelines.

B. HOCM with SAM (irrespective of MR severity) and LVOTO without significant septal hypertrophy and without a favourable anatomy for TASH

If there is a significant SAM phenomenon (with or without a redundant anterior mitral leaflet) combined with a mitral valve area exceeding 3.5–4 cm^2^, borderline LVOT hypertrophy, and not suitable coronary anatomy, primary M-TEER might be advantageous. This procedure can help lower the LVOT gradient and reduce MR.

C. HOCM with SAM-related MR and predominantly significant primary, mixed, or secondary MR

In our view, M-TEER emerged as a reasonable approach for patients with HOCM or those with hypertensive heart disease and concurrent primary mitral valve disease before performing TASH. In this cohort, those who develop a flail leaflet or mitral valve prolapse while in a long-term, stable anatomical and hemodynamic HOCM state will benefit from M-TEER, with TASH playing a complementary role in persistent symptoms. 

**Figure 4:**
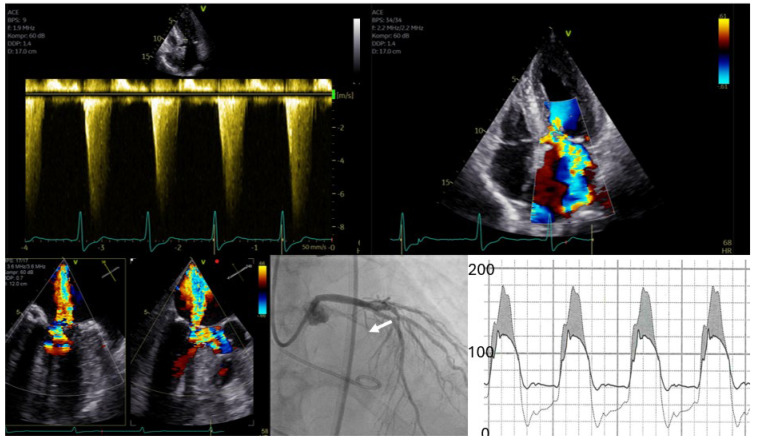
Hypertrophic obstructive cardiomyopathy with obstructive phenomena in the left ventricular outflow tract, with a peak velocity (Vmax) of about 7 m/s and systolic anterior motion of the mitral valve, resulting in an eccentric mitral regurgitation jet extending to the roof of the left atrium. (**left** and **right upper**, **left bottom**). A suitable first septal branch (white arrow) for TASH (**bottom-middle**). An increased rest gradient documented invasively (**right bottom**). (Courtesy of Ulrich Schäfer).

**Figure 5:**
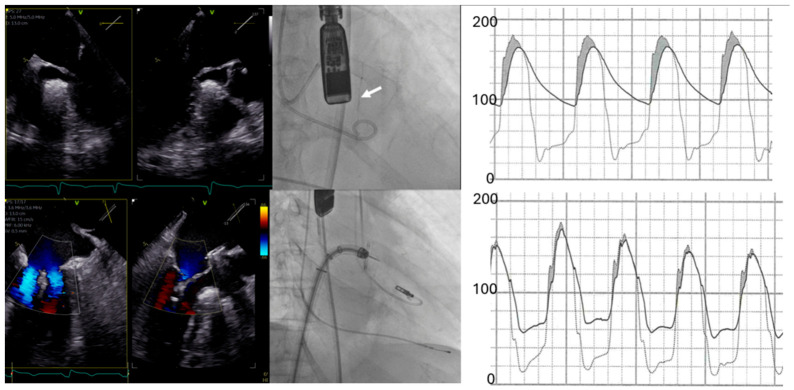
Successful TASH procedure with alcohol injection targeted at the first septal branch (white arrow, **top-middle**), after excluding the second and third septal branches as unsuitable, resulting in a substantial reduction of the LVOT gradient while the MR remained moderate to severe (**left** and **right upper**). In the same procedure, a single NT MitraClip (**bottom-middle**) was implanted in the A2/P2 segment, achieving near elimination of the MR and a residual LVOT gradient (**left** and **right bottom**). (Courtesy of Ulrich Schäfer). LVOT: left ventricular outflow tract, MR: mitral regurgitation, TASH: transcoronary ablation of septal hypertrophy.

If the primary mechanism of MR is the SAM with a high LVOT gradient, proceeding with TASH in patients with suitable coronary anatomy is appropriate. A stepwise approach, such as M-TEER, can be considered and implemented after a successful TASH, particularly when the LVOT gradient and SAM are eliminated, but moderate-to-severe secondary MR persists (Figure 4 and Figure 5). From our point of view, M-TEER should be considered as first-line treatment in the subgroup of patients suffering from coexisting severe secondary MR and disproportional lower LVOTO (maximal 30 mmHg or provoked gradient under 90 mmHg). If the LVOT gradient persists after M-TEER, TASH should be adopted to treat the remaining obstructive phenomena.

The choice between TASH and M-TEER should be tailored to the individual patient’s left ventricular morphology, considering echocardiographic and anatomical factors characteristics. When the cause of symptoms is unclear, and a complex, mixed mitral valve disease is present, a combined staged approach using both TASH and M-TEER may be beneficial for the patient’s symptoms. This strategy not only alleviates symptoms but also improves the patient’s hemodynamic status. Most patients with HOCM who underwent edge-to-edge repair benefited in terms of symptoms, and this was associated not only with MR reduction but also with near-complete elimination of the LVOT gradient. This alternative appears to work relatively well. According to published data, none of the patients underwent TASH after M-TEER, supporting this approach as relatively efficient. Nevertheless, it should keep the door open to using TASH in post–M–TEER patients with a significant residual LVOT gradient. A direct comparison study between M-TEER and TASH has not yet been conducted. More data are needed to answer key questions: which HOCM patient profile might benefit most from M-TEER; whether combining TASH and M-TEER offers superior results compared to monotherapy; when monotherapy is sufficient; what should be the first- and second-line percutaneous treatments; and how the LVOT gradient and mitral regurgitation behave over the long term in HOCM patients treated initially with M-TEER alone. 

## 5. Conclusions

Extensive experience with TASH suggests it is a first-line treatment for patients with HOCM and mild intrinsic mitral valve disease, especially SAM-related cases, rather than for those with a redundant anterior mitral leaflet or mild primary or secondary MR. M-TEER can be used as an alternative treatment for well-selected patients with HOCM, without primarily addressing the septal hypertrophy. A careful functional and anatomical assessment using transthoracic and transesophageal echocardiography, or even cardiac magnetic resonance imaging, is essential for identifying redundant anterior mitral valve leaflet. It should be emphasized that the presence of mitral valve redundancy can be the major pathomechanism of SAM-related mitral regurgitation and resultant obstruction, which can be targeted by primary M-TEER. M-TEER could eventually be incorporated into our treatment options for patients with borderline septal hypertrophy, severe primary or secondary MR, previous TASH failure, conduction abnormalities, absence of a suitable culprit septal branch, and echocardiographic features appropriate for M-TEER.

M-TEER is currently used off-label for patients with HOCM, with encouraging and promising results, although the available data come from case reports. Our enthusiasm for this no-touch septal technique should be supported subsequently by randomized controlled studies before it is adopted into our routine clinical practice.

## Figures and Tables

**Table 1:** Baseline characteristics.

Case	Age	Gender	EuroScore	NYHA	LVOT Obstruction: Resting Gradient, Provoked Gradient	IVSD	SAM	MR (Grade)	Medical Treatment	Previous Treatment
U. Schaefer [16], Case 1	69	Male	15.80%	III	36 mmHg, 136 mmHg	19 mm	Yes	Severe	Beta-blocker	Morrow and MVR two years before
U. Schaefer [16], Case 2	78	Female	30.30%	IV	75 mmHg, 155 mmHg	21 mm	Yes, but also mid-ventricular	Severe	Verapamil	TASH 6 months before
U. Schaefer [16], Case 3	76	Male	7.60%	III	84 mmHg, 145 mmHg	24 mm	Yes	Severe	Verapamil	PCI of LCMA with stent crossing the first septal branch
C. Kimmelstein [34], Case 1	68	Male	n.a.	III	39 mmHg, 88 mmHg	n.a.	Yes	Moderate to severe	Yes, n.a.	Failed TASH attempt (septal jailed with previous stent)
C. Kimmelstein [34], Case 2	66	Male	n.a.	III	80 mmHg, n.a.	n.a.	Yes	Severe	Yes, n.a.	Not suitable coronary anatomy
P. Sorajja [35], Case 1	87	Female	n.a.	III	61 mmHg, n.a.	18 mm	Yes	Severe	Negative inotropic agents	No
P. Sorajja [35], Case 2	90	Female	n.a.	III	44 mmHg, 81 mmHg	16 mm	Yes	Severe	Negative inotropic agents	No
P. Sorajja [35], Case 3	75	Male	n.a.	III	20 mmHg, 100 mmHg	17 mm	Yes	Severe	Negative inotropic agents	No
P. Sorajja [35], Case 4	72	female	n.a.	III	144 mmHg, not performed	23 mm	Yes	Severe	Negative inotropic agents	No
P. Sorajja [35], Case 5	89	Female	n.a.	III	36 mmHg, 92 mmHg	18 mm	Yes	Massive	Negative inotropic agents	No
A. Long [38], Case 1	72	Female	n.a.	III-IV	n.a., 94 mmHg	n.a.	Yes	Moderate to severe	Verapamil	No
A. Al Turk [39], Case 1	82	Male	n.a	n.a.	Not relevant, 120 mmHg	n.a.	Yes	Severe	n.a.	No
U. Schaefer [40], Case 1	69	Male	(logEUROscore 15.8%)	II-III	59–83 mmHg, >150 mmHg	n.a	Yes	Severe	n.a.	Surgical myectomy and mitral ring annuloplasty
S. Gupta [41], Case 1	76	Male	n.a.	III	74 mmHg (unknown if rest or provoked)	20 mm	Yes	Severe	n.a.	No
S. Gupta [41], Case 2	45	Male	n.a.	IV	85 mmHg (unknown if rest or provoked)	23 mm	Yes	Severe	n.a.	No
S. Gupta [41], Case 3	81	Female	n.a	III	130 mmHg (unknown if rest or provoked)	14 mm	Yes	Severe	n.a.	No
M. Coylewright [42], Case 1	84	Female	n.a.	II-III	20 mmHg, > 40 mmHg	n.a.	Yes	Severe, prolapse p2	n.a.	No
D. Harrison [43], Case 1	26	Male	n.a.	IV	95 mmHg, n.a.	n.a.	Yes	Severe	n.a.	Surgical myectomy
N. Wong [44], Case 1	76	Female	7.64%	III	76 mmHg, n.a.	23 mm	Yes	Severe (Flail P2)	Verapamil and beta-blocker	No
X. Huang [45], Case 1	68	Female	n.a.	II-III	107 mmHg (unknown if rest or provoked)	15.2 mm	Yes	Severe (degenerative A2/A3)	n.a.	No
J. Rezkalla [46], Case 1	71	Male	n.a.	II-III	n.a., 150 mmHg (dobutamine)	n.a.	Yes	Dynamic severe	Beta-blocker	TASH (2015), surgical myectomy (2021)
O. Rabi [47], Case 1	53	Female	n.a.	IV	154 mmHg (unknown if rest or provoked)	24 mm	Yes	Severe	Beta-blocker	No
C. Bourque [48], Case 1	68	Male	n.a.	III	40 mmHg (unknown if rest or provoked)	n.a.	Yes, acute	Severe dynamic annular dilatation	n.a.	No
A. Pantazis [49], Case 1	n.a.	n.a.	n.a.	n.a.	n.a.	n.a.	n.a.	Moderate to severe	n.a.	n.a.
A. Pantazis [49], Case 2	n.a.	n.a.	n.a.	n.a.	n.a.	n.a.	n.a.	Severe	n.a.	n.a.
A. Pantazis [49], Case 3	n.a.	n.a.	n.a.	n.a.	n.a.	n.a.	n.a.	Severe	n.a.	n.a.
A. Pantazis [49], Case 4	n.a.	n.a.	n.a.	n.a.	n.a.	n.a.	n.a.	Moderate to severe	n.a.	n.a.
A. Pantazis [49], Case 5	n.a.	n.a.	n.a.	n.a.	n.a.	n.a.	n.a.	Severe	n.a.	n.a.
A. Pantazis [49], Case 6	n.a.	n.a.	n.a.	n.a.	n.a.	n.a.	n.a.	Moderate to severe	n.a.	n.a.

IVSD: intraventricular septal interventricular septum thickness at end-diastole, LCMA: left coronary main artery, LVOT: left ventricular outflow tract, MR: mitral regurgitation, MVR: mitral valve reconstruction, n.a.: not available, PCI: percutaneous coronary intervention, NYHA: New York Heart Association, SAM: systolic anterior motion, TASH: transcoronary ablation of septal hypertrophy.

**Table 2:** Post MitraClip.

Case	MR Grade	MV Gradient	LVOT Gradient	Follow Up	Asystole, Av Block	Pacemaker/AICD
U. Schaefer [16], Case 1	Trace	5 mmHg	3 mmHg	Six weeks later: trace MR, reduced LVOT gradient, NYHA I-II	No	No
U. Schaefer [16], Case 2	Trace	3 mmHg	7 mmHg	Six weeks later: trace MR, reduced LVOT gradient, NYHA I-II	No	No
U. Schaefer [16], Case 3	Trace	3 mmHg	13 mmHg	Six weeks later: trace MR, reduced LVOT gradient, NYHA I-II	No	No
C. Kimmelstein [34], Case 1	Trace	3 mmHg	No obstruction	1 and 2 months later: NYHA I	No	No
C. Kimmelstein [34], Case 2	Trace	4 mmHg	No obstruction	1 and 2 months later: NYHA I	No	No
P. Sorajja [35], Case 1	Mild	6 mmHg	17 mmHg	19 months later: mild MR, PGmean 8 mmHg, Vmax 5.2 m/s, NYHA II	No	No
P. Sorajja [35], Case 2	Mild	2 mmHg	3 mmHg	16 months later: trace MR, PGmean 3 mmHg, Vmax 0.8 m/s, NYHA I	No	No
P. Sorajja [35], Case 3	Trace	3 mmHg	13 mmHg	12 months later: trace MR, PGmean 3 mmHg, Vmax 5.1 m/s, NYHA I	No	No
P. Sorajja [35], Case 4	Mild	4 mmHg	10 mmHg	16 months later: mild MR, PGmean 5 mmHg, Vmax. 6.2 m/s, NYHA II	No	No
P.Sorajja [35], Case 5	Mild	3 mmHg	16 mmHg	10 months later: mild MR, PGmean 3 mmHg, Vmax. 1.9 m/s, NYHA I	No	No
A. Long [38], Case 1	Mild	5 mmHg	27 mmHg	1 month later: mild MR, PGmean 5 mmHg, LVOT gradient at rest/provoked 27 mmHg, no evidence of SAM	No	No
A. Al Turk [39], Case 1	Trace	n.a.	<5 mmHg	n.a. symptom resolution	No	No
U. Schaefer [40], Case 1	Mild	3 mmHg	No obstruction	12 months later: mild MR, no relevant gradient, NYHA I	No	No
S. Gupta [41], Case 1	Mild	5 mmHg	13 mmHg	n.a.	No	No
S. Gupta [41], Case 2	Mild	4 mmHg	12 mmHg	n.a.	No	No
S. Gupta [41], Case 3	Mild	4 mmHg	10 mmHg	n.a.	No	No
M. Coylewright [42], Case 1	Moderate	6 mmHg	No obstruction	n.a.	No	No
D. Harrison [43], Case 1	Moderate	n.a.	10 mmHg	n.a.	No	No
N. Wong [44], Case 1	Mild to moderate	n.a.	33 mmHg	1 and 6 months later: moderate MR, LVOT gradient 38 mmHg, symptoms improved	No	No
X. Huang [45], Case 1	Trace	n.a.	13 mmHg	n.a.	No	No
J. Rezkalla [46], Case 1	Mild	5 mmHg	Complete resolution	1 month later: no MR, without LVOT Gradient, NYHA I	No	No
O. Rabi [47], Case 1	Reduced	n.a.	90 mmHg	1 month later: moderate to severe MR, PGmean 7 mmHg, LVOT gradient 29 mmHg, NYHA I-II	No	No
C. Bourque [48], Case 1	Moderate	n.a.	n.a.	6 months later: MR mild, symptoms improved	No	Yes
A. Pantazis [49], Case 1	Mild	n.a.	n.a.	12 months later: no MR recurrence	No	No
A. Pantazis [49], Case 2	Moderate	n.a.	n.a.	12 months later: no MR recurrence	No	No
A. Pantazis [49], Case 3	Moderate to severe	n.a.	n.a.	1 month later: MR recurrence	No	No
A. Pantazis [49], Case 4	Mild	n.a.	n.a.	8 months later: MR recurrence	No	No
A. Pantazis [49], Case 5	Mild	n.a.	n.a.	12 months later: no MR recurrence	No	No
A. Pantazis [49], Case 6	Mild	n.a.	n.a.	12 months later: no MR recurrence	No	No

AICD: automatic implantable cardioverter-defibrillator, Av-block: atrioventricular block, LVOT: left ventricular outflow tract, MR: mitral regurgitation, MV: mitral valve, n.a.: not available, NYHA: New York Heart Association.

## Data Availability

The data presented in this study are available on reasonable request from the corresponding author.

## Abbreviations

The following abbreviations are used in this manuscript:

AV block	Atrioventricular block
HCM	Hypertrophic cardiomyopathy
HOCM	Hypertrophic obstructive cardiomyopathy
LBBB	Left bundle branch block
LVOT	Left ventricular outflow tract
LVOTO	Left ventricular outflow tract obstruction
MR	Mitral regurgitation
M-TEER	Mitral transcatheter edge to edge repair
NYHA	New York Heart Association
SAM	Systolic anterior motion
SESAME	Septal Scoring Along Midline Endocardium
SLDA	Single leaflet device attachment
TASH	Transcoronary ablation of septal hypertrophy

## References

[1] Massera D., Sherrid M.V., Maron M.S., Rowin E.J., Maron B.J. (2023). How common is hypertrophic cardiomyopathy… really?: Disease prevalence revisited 27 years after CARDIA. Int. J. Cardiol..

[2] Maron M.S., Olivotto I., Zenovich A.G., Link M.S., Pandian N.G., Kuvin J.T., Nistri S., Cecchi F., Udelson J.E., Maron B.J. (2006). Hypertrophic cardiomyopathy is predominantly a disease of left ventricular outflow tract obstruction. Circulation.

[3] Ommen S.R., Mital S., Burke M.A., Day S.M., Deswal A., Elliott P., Evanovich L.L., Hung J., Joglar J.A., Kantor P. (2020). 2020 AHA/ACC Guideline for the Diagnosis and Treatment of Patients with Hypertrophic Cardiomyopathy: A Report of the American College of Cardiology/American Heart Association Joint Committee on Clinical Practice Guidelines. Circulation.

[4] Maron B.J., Desai M.Y., Nishimura R.A., Spirito P., Rakowski H., Towbin J.A., Rowin E.J., Maron M.S., Sherrid M.V. (2022). Diagnosis and Evaluation of Hypertrophic Cardiomyopathy: JACC State-of-the-Art Review. J. Am. Coll. Cardiol..

[5] Arbelo E., Protonotarios A., Gimeno J.R., Arbustini E., Barriales-Villa R., Basso C., Bezzina C.R., Biagini E., Blom N.A., de Boer R.A. (2023). 2023 ESC Guidelines for the management of cardiomyopathies: Developed by the task force on the management of cardiomyopathies of the European Society of Cardiology (ESC). Eur. Heart J..

[6] Ommen S.R., Ho C.Y., Asif I.M., Balaji S., Burke M.A., Day S.M., Dearani J.A., Epps K.C., Evanovich L., Ferrari V.A. (2024). 2024 AHA/ACC/AMSSM/HRS/PACES/SCMR Guideline for the Management of Hypertrophic Cardiomyopathy: A Report of the American Heart Association/American College of Cardiology Joint Committee on Clinical Practice Guidelines. Circulation.

[7] Hang D., Schaff H.V., Nishimura R.A., Lahr B.D., Abel M.D., Dearani J.A., Ommen S.R. (2019). Accuracy of Jet Direction on Doppler Echocardiography in Identifying the Etiology of Mitral Regurgitation in Obstructive Hypertrophic Cardiomyopathy. J. Am. Soc. Echocardiogr..

[8] Troy A.L., Narula N., Massera D., Adlestein E., Alvarez I.C., Janssen P.M.L., Moreira A.L., Olivotto I., Stepanovic A., Thomas K. (2023). Histopathology of the Mitral Valve Residual Leaflet in Obstructive Hypertrophic Cardiomyopathy. JACC Adv..

[9] Woo A., Jedrzkiewicz S. (2011). The mitral valve in hypertrophic cardiomyopathy: It’s a long story. Circulation.

[10] Boissier F., Achkouty G., Bruneval P., Fabiani J.N., Nguyen A.T., Riant E., Desnos M., Hagège A. (2015). Rupture of mitral valve chordae in hypertrophic cardiomyopathy. Arch. Cardiovasc. Dis..

[11] Silbiger J.J. (2016). Abnormalities of the Mitral Apparatus in Hypertrophic Cardiomyopathy: Echocardiographic, Pathophysiologic, and Surgical Insights. J. Am. Soc. Echocardiogr..

[12] Anwar A.M., Soliman O.I., Nemes A., Germans T., Krenning B.J., Geleijnse M.L., Van Rossum A.C., ten Cate F.J. (2007). Assessment of mitral annulus size and function by real-time 3-dimensional echocardiography in cardiomyopathy: Comparison with magnetic resonance imaging. J. Am. Soc. Echocardiogr..

[13] Molisana M., Selimi A., Gizzi G., D’Agostino S., Ianni U., Parato V.M. (2022). Different mechanisms of mitral regurgitation in hypertrophic cardiomyopathy: A clinical case and literature review. Front. Cardiovasc. Med..

[14] Hausleiter J., Stocker T.J., Adamo M., Karam N., Swaans M.J., Praz F. (2023). Mitral valve transcatheter edge-to-edge repair. EuroIntervention.

[15] Praz F., Borger M.A., Lanz J., Marin-Cuartas M., Abreu A., Adamo M., Marsan N.A., Barili F., Bonaros N., Cosyns B. (2026). 2025 ESC/EACTS Guidelines for the management of valvular heart disease: Developed by the task force for the management of valvular heart disease of the European Society of Cardiology (ESC) and the European Association for Cardio-Thoracic Surgery (EACTS). Eur. Heart J..

[16] Schäfer U., Frerker C., Thielsen T., Schewel D., Bader R., Kuck K.H., Kreidel F. (2015). Targeting systolic anterior motion and left ventricular outflow tract obstruction in hypertrophic obstructed cardiomyopathy with a MitraClip. EuroIntervention.

[17] Liebregts M., Vriesendorp P.A., Mahmoodi B.K., Schinkel A.F., Michels M., ten Berg J.M. (2015). A Systematic Review and Meta-Analysis of Long-Term Outcomes After Septal Reduction Therapy in Patients with Hypertrophic Cardiomyopathy. JACC Heart Fail..

[18] Maron B.J., Nishimura R.A. (2014). Revisiting arrhythmic risk after alcohol septal ablation: Is the pendulum finally swinging … back to myectomy?. JACC Heart Fail..

[19] Talreja D.R., Nishimura R.A., Edwards W.D., Valeti U.S., Ommen S.R., Tajik A.J., Dearani J.A., Schaff H.V., Holmes D.R. (2004). Alcohol septal ablation versus surgical septal myectomy: Comparison of effects on atrioventricular conduction tissue. J. Am. Coll. Cardiol..

[20] Quintana E., Bajona P., Arguis M.J., Prat-González S. (2017). Septal myectomy after failed septal alcohol ablation. Ann. Cardiothorac. Surg..

[21] Eggebrecht H., Schelle S., Puls M., Plicht B., von Bardeleben R.S., Butter C., May A.E., Lubos E., Boekstegers P., Ouarrak T. (2015). Risk and outcomes of complications during and after MitraClip implantation: Experience in 828 patients from the German TRAnscatheter mitral valve interventions (TRAMI) registry. Catheter. Cardiovasc. Interv..

[22] Schnitzler K., Hell M., Geyer M., Kreidel F., Münzel T., von Bardeleben R.S. (2021). Complications Following MitraClip Implantation. Curr. Cardiol. Rep..

[23] Wang T.K.M., Chatfield A., Wang M.T.M., Ruygrok P. (2020). Comparison of percutaneous MitraClip versus mitral valve surgery for severe mitral regurgitation: A meta-analysis: Mitraclip and mitral valve surgery meta-analysis. AsiaIntervention.

[24] Cameron J., Sutherland N., Chow C., Han H., Yudi M., Sabbag A., Raman J., Sanders P., Farouque O., Lim H. (2025). Ventricular Arrhythmia and Sudden Death Outcomes Before and After Percutaneous Valve Repair for Mitral Regurgitation. Heart Lung Circ..

[25] Verma B.R., Shekhar S., Isogai T., Chava R., Raeisi-Giglou P., Bansal A., Khubber S., Montane B., Vaidya P., Kaur S. (2022). Postdischarge-to-30-Day Mortality Among Patients Receiving MitraClip: A Systematic Review and Meta-Analysis. Struct. Heart.

[26] Pelliccia F., Niccoli G., Gragnano F., Limongelli G., Moscarella E., Andò G., Esposito A., Stabile E., Ussia G.P., Tarantini G. (2019). Alcohol septal ablation for hypertrophic obstructive cardiomyopathy: A contemporary reappraisal. EuroIntervention.

[27] Veselka J., Jensen M., Liebregts M., Cooper R.M., Januska J., Kashtanov M., Dabrowski M., Hansen P.R., Seggewiss H., Hansvenclova E. (2020). Alcohol septal ablation in patients with severe septal hypertrophy. Heart.

[28] Angelini P. (2007). The “1st septal unit” in hypertrophic obstructive cardiomyopathy: A newly recognized anatomo-functional entity, identified during recent alcohol septal ablation experience. Tex. Heart Inst. J..

[29] Lawrenz T., Lieder F., Bartelsmeier M., Leuner C., Borchert B., Meyer zu Vilsendorf D., Strunk-Mueller C., Reinhardt J., Feuchtl A., Stellbrink C. (2007). Predictors of complete heart block after transcoronary ablation of septal hypertrophy: Results of a prospective electrophysiological investigation in 172 patients with hypertrophic obstructive cardiomyopathy. J. Am. Coll. Cardiol..

[30] Alam M., Dokainish H., Lakkis N.M. (2009). Hypertrophic obstructive cardiomyopathy-alcohol septal ablation vs. myectomy: A meta-analysis. Eur. Heart J..

[31] Quintana E., Sabate-Rotes A., Maleszewski J.J., Ommen S.R., Nishimura R.A., Dearani J.A., Schaff H.V. (2015). Septal myectomy after failed alcohol ablation: Does previous percutaneous intervention compromise outcomes of myectomy?. J. Thorac. Cardiovasc. Surg..

[32] Greenbaum A.B., Ueyama H.A., Gleason P.T., Khan J.M., Bruce C.G., Halaby R.N., Rogers T., Hanzel G.S., Xie J.X., Byku I. (2024). Transcatheter Myotomy to Reduce Left Ventricular Outflow Obstruction. J. Am. Coll. Cardiol..

[33] McCabe J.M., Newton S., Danek B.A., Elison D., Chung C.J., Sheu R., Jelacic S., Condos G.J., Canovas E., Greenbaum A.B. (2025). SESAME technique: Septal scoring along the midline endocardium. EuroIntervention.

[34] Kimmelstiel C., Everett K.D., Jain P., Miyashita S., Botto R., Resor C., Rowin E., Maron M., Kapur N.K. (2022). Transcatheter Mitral Intervention Relieves Dynamic Outflow Obstruction and Reduces Cardiac Workload in Hypertrophic Cardiomyopathy. Circ. Heart Fail..

[35] Sorajja P., Pedersen W.A., Bae R., Lesser J.R., Jay D., Lin D., Harris K., Maron B.J. (2016). First Experience With Percutaneous Mitral Valve Plication as Primary Therapy for Symptomatic Obstructive Hypertrophic Cardiomyopathy. J. Am. Coll. Cardiol..

[36] Thomas F., Rader F., Siegel R.J. (2017). The Use of MitraClip for Symptomatic Patients with Hypertrophic Obstructive Cardiomyopathy. Cardiology.

[37] Mascarenhas L., Yang G., Sharma A., Bertog S., Hubers S., Adabag S. (2025). Outcomes of Transcatheter Edge-to-Edge Mitral Valve Repair in Hypertrophic Cardiomyopathy: A Patient-Level Meta-Analysis. Struct. Heart.

[38] Long A., Mahoney P. (2020). Use of MitraClip to Target Obstructive SAM in Severe Diffuse-Type Hypertrophic Cardiomyopathy: Case Report and Review of Literature. J. Invasive Cardiol..

[39] Al Turk A.A., Ibrahim A.W., Eng M.H. (2023). Acute Hemodynamic Impact of Transcatheter Edge-to-Edge Repair in Hypertrophic Cardiomyopathy. J. Soc. Cardiovasc. Angiogr. Interv..

[40] Schäfer U., Kreidel F., Frerker C. (2014). MitraClip implantation as a new treatment strategy against systolic anterior motion-induced outflow tract obstruction in hypertrophic obstructive cardiomyopathy. Heart Lung Circ..

[41] Gupta S., Slater M., Heitner S., Wei K. (2016). TCT-810 Percutaneous Mitral Valve Repair for Management of Systolic Anterior Motion and Mitral Regurgitation Associated with Hypertrophic Cardiomyopathy. J. Am. Coll. Cardiol..

[42] Coylewright M., O’Neill E.S., Robb J.F., McCullough J.N., Tighe C.M., Callahan J.M., Beaver T.A. (2017). Reduction of left ventricular outflow tract obstruction with transcatheter mitral valve repair. Echocardiography.

[43] Harrison D., Montano S., Kessler W., Vakamudi S., Gajjar M.M., Cauthen C., Pirwitz M.J. (2022). An off-label lifesaver: Transcatheter mitral valve repair for severe mitral regurgitation associated with hypertrophic cardiomyopathy. J. Am. Coll. Cardiol..

[44] Wong N., Hamid N., Tang H.C., Yeo K.K. (2018). Killing two birds with one stone-MitraClip for flail P2 and systolic anterior motion of mitral valve: A case report. Eur. Heart J. Case Rep..

[45] Huang X., Sung S.H., Su M., Wang Y. (2023). One stone hits two birds: Transcatheter mitral valve edge-to-edge repair for hypertrophic obstructive cardiomyopathy and severe mitral regurgitation. Eur. Heart J..

[46] Rezkalla J., Eleid M.F. (2023). Transcatheter Treatment of Residual Systolic-Anterior Motion and Severe Mitral Regurgitation in Hypertrophic Cardiomyopathy. JACC Cardiovasc. Interv..

[47] Rabi O., Carasso S., Helviz Y., Karmi M., Levin P., Shuvy M. (2025). Rescue Mitral Clip Therapy in Hypertrophic Obstructive Cardiomyopathy with Severe Mitral Regurgitation and Combined Shock. CJC Open.

[48] Bourque C., Dijos M., Leroux L., Labrousse L., Metras A., Michaud M., Hébert M., Réant P., Lafitte S. (2020). Eclipsed Functional Mitral Regurgitation Destabilizing Hypertrophic Cardiomyopathy: An Unusual Case Treated with MitraClip. CJC Open.

[49] Pantazis A., Cheang M.H., Mullen M., Elliott P., Mckenna W., Aggarwal S.K., TomeEsteban M.T., Reinthaler M., Delahunty N. (2014). 94 Percutaneous Mitral Repair in Hypertrophic Cardiomyopathy. Heart.

[50] Sorajja P., Binder J., Nishimura R.A., Holmes D.R., Rihal C.S., Gersh B.J., Bresnahan J.F., Ommen S.R. (2013). Predictors of an optimal clinical outcome with alcohol septal ablation for obstructive hypertrophic cardiomyopathy. Catheter. Cardiovasc. Interv..

[51] Valeti U.S., Nishimura R.A., Holmes D.R., Araoz P.A., Glockner J.F., Breen J.F., Ommen S.R., Gersh B.J., Tajik A.J., Rihal C.S. (2007). Comparison of surgical septal myectomy and alcohol septal ablation with cardiac magnetic resonance imaging in patients with hypertrophic obstructive cardiomyopathy. J. Am. Coll. Cardiol..

[52] Lu D.Y., Hailesealassie B., Ventoulis I., Liu H., Liang H.Y., Nowbar A., Pozios I., Canepa M., Cresswell K., Luo H.C. (2017). Impact of peak provoked left ventricular outflow tract gradients on clinical outcomes in hypertrophic cardiomyopathy. Int. J. Cardiol..

[53] Faza N.N., Chebrolu L.B., El-Tallawi K.C., Zoghbi W.A. (2022). An Integrative, Multiparametric Approach to Mitral Regurgitation Evaluation: A Case-Based Illustration. JACC Case Rep..

